# A New Perspective on Sexual Mixing among Men Who Have Sex with Men by Body Image

**DOI:** 10.1371/journal.pone.0113791

**Published:** 2014-11-20

**Authors:** Ka-Kit Leung, Horas T. H. Wong, Claire M. Naftalin, Shui Shan Lee

**Affiliations:** Stanley Ho Centre for Emerging Infectious Diseases, The Chinese University of Hong Kong, Hong Kong, China; Alberta Provincial Laboratory for Public Health/University of Alberta, Canada

## Abstract

**Background:**

“Casual sex” is seldom as non-selective and random as it may sound. During each sexual encounter, people consciously and unconsciously seek their casual sex partners according to different attributes. Influential to a sexual network, research focusing on quantifying the effects of physical appearance on sexual network has been sparse.

**Methods:**

We evaluated the application of Log odds score (LOD) to assess the mixing patterns of 326 men who have sex with men (MSM) in Hong Kong in their networking of casual sex partners by Body Image Type (BIT). This involved an analysis of 1,196 respondents-casual sex partner pairs. Seven BITs were used in the study: *Bear*, *Chubby*, *Slender*, *Lean toned*, *Muscular*, *Average* and *Other*.

**Results:**

A hierarchical pattern was observed in the preference of MSM for casual sex partners by the latter's BIT. Overall, *Muscular* men were most preferred, followed by *Lean toned* while the least preferred was *Slender*, as illustrated by LOD going down along the hierarchy in the same direction. Marked avoidance was found between men who self-identified as *Chubby* and men of *Other* body type (within-group-LOD: 1.25–2.89; between-group-LOD: <−1). None of the respondents reported to have networked a man who self-identified as *Average* for casual sex.

**Conclusions:**

We have demonstrated the possibility of adopting a mathematical prototype to investigate the influence of BIT in a sexual network of MSM. Construction of matrix based on culture-specific BIT and cross-cultural comparisons would generate new knowledge on the mixing behaviors of MSM.

## Introduction

Physical appearance is an important factor that affects human sexual behaviors [1]. Corporal attractiveness strongly impacts first impressions and encourages individual's self-disclosure, which in turn opens up opportunities for intimate relationships [2]–[4]. Indeed, the relationships of physical appearance and sexuality have been widely studied. Most reports have suggested that, regardless of gender or sexuality, human engagement in consensual sexual relationships is somehow influenced by the way individuals look and how they perceive their bodies [5]–[8]. Sex, especially casual relationship, is therefore seldom as non-selective and random as it may sound. Apparently, people consciously and unconsciously seek their casual sex partners according to different attributes. Epidemiologists and network scientists have, however, been unable to construct a robust mathematical model to explore and simulate the effects of body appearance in sexual networks as quantitative investigations in this area were insufficient.

Previous research has described the heterogeneity of the gay/bisexual community (referred as men who have sex with men (MSM) in public health terminology) and the self-segregation among different gay sub-cultures [9]. The social and sexual networking among different subgroups of MSM could be quite discrete [10]. One salient example is the *Bear* community of which men are often described as having a physical body with masculine attributes such as being stocky and hairy [11], [12]. A survey on the *Bear* community in the United States has suggested the *Bears*' preference for partners of similar somatotype and their rejection of non-bearish sex partners [13]. Likewise, the *Bear* body has been perceived as less attractive by the “mainstream” gay men who embrace thinner, younger and less hairy body ideals [11], [12].

This discreteness in networking among MSM provides a unique model for the development of a mathematical prototype to assess how physical appearance is embedded in a sexual network. In this study, we set out to construct a mixing matrix by body image types (BIT) in an MSM network by using log odds score (LOD) to assess the likelihood of respondent-partner pair. LOD is the logarithm of the likelihood of an event relative to its likelihood under a null model. The concept has been widely used in diverse disciplines, to assess the statistical significance of a pattern in Biological and Biomedical Sciences [14], [15], Chemistry [16], Economics [17] and Geophysics [18].

## Materials and Methods

### Ethics Statement

This survey study was reviewed and approved by the Survey and Behavioral Research Ethics Committee of the Chinese University of Hong Kong. All participants have given informed consent prior to the study.

### Participant enrollment

The study was originally designed to explore the network and HIV transmission risk among MSM in Hong Kong. Key study domains of the study included socio-demographics, patterns of sexual networking and safer sex practices with both casual and regular partners. The methodological details and results of the study have been presented elsewhere [19]. Briefly, to minimize sampling bias due to affiliation with physical venues [20], MSM were conveniently recruited at 18 different saunas, from December 2010 to January 2011. The participants recruited in the original study [19] were also asked to provide body image type (BIT) information of themselves and of their recent sex partners.

### Body image type (BIT) mixing pattern assessment

We attempted to use data derived from the study to identify the direction (seeking and being sought) of mixing pattern by BIT. The term “Body image” is deliberately used here because it reflects the respondent's perception towards their body appearances, rather than a true body figure that could be objectively measured. BIT was assessed by asking the respondents to choose from a list of categorical terms that best describe themselves and the 5 most recent sex partners they had sought in the preceding 3 months [19]. The terms used were originally phrased in Cantonese (commonest Chinese dialect in Hong Kong), saliently proclaimed by MSM informants during previous field studies. They included *hung-zuk* (Bears), *fei-bun* (fat/chubby), *sau* (thin/slender/skinny), *git-sat* (lean toned/firmed body), *gei-juk-jing* (muscular), *jat-bun* (normal/average) and *other*. Respondents could choose as many terms as they found appropriate. They were allowed to opt-out by choosing “not defined/I don't know/I don't like labels”. The final list of BITs used for analysis were *Bear* (B), *Chubby* (C), *Slender* (S), *Lean toned* (L), *Muscular* (M), *Average* (A) and *Other* (O).

To achieve our objectives, we first counted the number of BITs for respondents and the casual sex partners (one-night-stand partners) sought by the respondents. Our logic of focusing on the BIT of “casual” partners was that we believed physical appearance has a more profound role in casual sex engagement than in sex with regular partners. In regular sexual relationships (such as emotionally-attached or “friends-with-benefits” relationships), other intangible factors such as trust, emotion or level of intimacy may blur the effect of physical appearance [21], [22]. Observed and expected frequency for each respondent-partner BIT pair was then calculated. The extent of mixing was expressed as a log odds ratio of an observed frequency for a respondent-partner BIT pair over the product of the two expected frequencies of each BIT involved. The score, i.e. LOD, carried a sign (positive indicating preference while negative indicating avoidance) while the magnitude was assumed to reflect the concordance and intensity of the mixing. A value of 0.01 was added in cases of 0 for adjustment.

To assess the mixing patterns between MSM of different BITs, we constructed a mixing matrix in a way analogous to the construction of substitution matrix in bioinformatics and evolutionary biology. The number of times respondents of BIT *i* having casual sex with partners of BIT *j* was tallied, denoted by q_ij_. Different from the substitution matrix that is symmetric, direction matters in mixing matrix. In other words, q_ij_ is not necessarily the same as q_ji_. The observed probability p_ij_ of respondents of BIT *i* having casual sex with partners of BIT *j* is then obtained by:
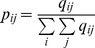
Accordingly, the marginal probability p_i_, the expected probability of occurrence of BIT *i* can be calculated by:
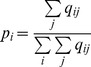
The log odds score (LOD) is defined as:
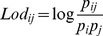



## Results and Discussions

A total of 326 MSM were recruited in the two-month study period. All participants were Chinese except two who had missing ethnicity data. MSM indicated their own and their partner's BIT, the latter involving a total of 1579 casual partners. Only those 1196 respondent-partner pairs with BIT were included in the analysis. In this study, we have applied a new mathematical method to investigate the influence of BIT in a sexual network of MSM. The statistics of the respondents are summarized in Table 1. Kruskal Wallis test shows no statistically significant difference among MSM of different BIT in terms of education (p = 0.81) and age (p = 0.20)

**Table 1:** Distribution of education and age of respondents by Body Image Type (BIT).

	Education (Number)				Age (Median)
BIT*	Primary	Secondary	Post-secondary or above	Unknown	
B		3	4		35
C		16	14	1	34
S	1	37	58		27
L	1	34	72		29
M		17	29		32
A		19	24	1	32
O		50	83	2	31
Subtotal	2	176	284	4	

*Abbreviations: *Bear* (B), *Chubby* (C), *Slender* (S), *Lean toned* (L), *Muscular* (M), *Average* (A) and *Others* (O).

**Figure 1:**
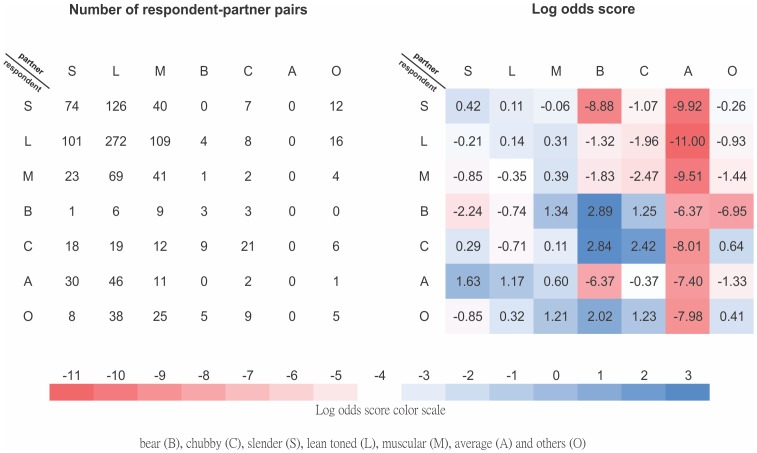
Matrix for MSM mixing by Body Image Type (BIT). The left and right panels demonstrate the number and LOD respectively. The colors represent preference (blue) and avoidance (red). Intensity is represented by the tone of the colors (the darker the more intense).

The most prevalent respondent-partner BIT pairs were the *Lean toned-Lean toned* (L-L) pairs (22.7%), *Slender-Lean toned* (S-L) pairs (10.5%), *Lean toned-Muscular* (L-M) pairs (9.1%) and *Lean toned-Slender* (L-S) pairs (8.4%). All the 90 type A related pairs identified were from type A respondents and none of these respondents reported to have sought a sex partner of Type A. Self-BIT preference was observed in all BITs except Type A (LOD A-A = −7.4). The strongest BIT preference was found in Types B and C, with LOD B-B = 2.89, LOD C-C = 2.42, LOD C-B = 2.84, and it appeared that respondents of Type B were much less likely to seek a sex partner of Type C than the other way round (LOD B-C = 1.25). (Figure 1)

Although none of our respondents had sought a Type A partner (adjusted LOD were all <−6), Type A respondents preferred partners of other types (LOD range: 0.60–1.63) except Type C (LOD A-C = −0.37) and B (LOD A-B = −6.37). The group of Types S, L and M and the group of Types B and C were rather mutually exclusive, as demonstrated by the negative or small positive LOD (range: −8.88 to 1.34). Despite this, respondents of Types S, L and M seemed less likely to seek a Type B or C partner (LOD range: −8.88 to −1.07) than the reverse (LOD range: −2.24 to 1.34). Type M appeared to be a “sink”, as the individuals were almost universally preferred by all types. However, they also exhibited universal low preference for other types (LOD range: −9.51 to 0.35), including themselves (LOD M-M = 0.39). (Figure 1) Our findings echo previous data that suggested the heterogeneity of MSM community based on physical appearance. Characteristically, men with bigger body size (*Bear/Chubby*) belong to a unique and independent subgroup within the entire MSM community [11], [12]. In our study, the sole preference for “mainstream” (*Slender*, *Lean toned* or *Muscular*) men of the *Average* respondents has further isolated the *Bear/Chubby* community.

Interestingly a hierarchical pattern can be observed in the sexual networks of our respondents – at the topmost level were *Muscular* men (LOD M-S = −0.85; LOD M-L = −0.35), followed by *Lean toned* men (LOD L-S = −0.21) while *Slender* men were at the lowest level. Indeed, studies in gender and body image have already illustrated the predilection for muscular men in the gay community [11], [23]–[25]. Under Connell's proposed paradigm of hegemonic masculinity, gay masculinities are socially perceived as a subordinate form of masculinity due to heterosexuality and homophobia [26]. At the same time, gay men have been subjected to the authority in hegemonic masculinity in their lives which shapes their perceptions of gayness [27]. Therefore, masculine gay men may in fact embody the stereotypical traits of traditional masculinity ideology [28]. As Connell wrote, ‘the choice of a man as a sexual object is not just the choice of a body-with-penis, it is the choice of embodied masculinity’ [27], gay men are attracted to physiques that symbolize a higher level of masculinity (such as being muscular and athletic), while the value of less masculine bodies (such as feminine, slender and boney) is sometimes debased [29]. This may also explain the hierarchical pattern observed in this study. Moreover, this craving for masculine (or sometimes hyper-masculine) bodies is more prominent in the *Bear* communities [11], a phenomenon which is also confirmed in our results.

One most puzzling finding here is that *Average* MSM appears to act as a source in the sexual relationships. Some respondents described themselves as *Average* but they classified none of their partners as *Average*. One possible explanation is the effect of recall bias, as respondents might opt to highlight the more prominent characteristics of their partners, rather than just identifying their partners as *Average*. Another explanation is that in sex-on-premise venues like gay saunas where different types of potential sex partners are abundant, *Average* men may be less popular than *Muscular* or *Lean toned* men. Therefore one has to actively seek a sex partner rather than being sought passively. Future ethnographic studies in gay saunas may provide a better insight for confirming the existence of the phenomenon.

In Hong Kong, there have been no formal studies on the BIT of MSM in Hong Kong. Their categorization could however be reflected from local gay websites, magazines and social venues. In his book on Chinese homosexuality, Kong mentioned the disentanglement of *Bears* from the hegemonic gay population in Hong Kong. There are saunas and pubs that specifically target *Bears* in the region [30]. We are thus confident to hypothesize that sub-cultures and self-segregation among gay men do exist in Hong Kong. As the BIT used in this study were proclaimed by local informants during previous field studies, it should be noted that the categorization is socially and culturally constructed, which may not necessarily be translated to communities outside Hong Kong. Indeed, body images and sexual desirability are highly dynamic under changing social environments. Studies of psychology and psychiatry have demonstrated the tendency of gay men to experience poor body image and a larger drive to thinness [23], but this might not be true among non-Western societies [31]. The formation of gay subculture and the identity may also be different from a Chinese emic perspective [32]. However, exploring their true meanings and definitions require anthropological and ethnographical efforts and was not the aim of this analysis.

Finally, it must be cautioned that our data were drawn from egocentric networks and thus the full network configuration could not be uncovered. Also, we did not adjust for residential status, that could have confounded the mixing pattern among MSM [33]. Nevertheless, our analytic approach has provided a novel perspective to understand the basic components of sexual networks and the potential drivers of network dynamics. We have added new knowledge for evaluating respondent driven sampling by proposing a method which may be adapted to quantify the likelihood of a seed to recruit partners of the same or different BIT. To conclude, we have demonstrated the possibility to adopt a mathematical prototype, which was not previously used in network analysis, to explore the influence of BIT in a sexual network of MSM. Construction of mixing matrix based on culture-specific BIT and subsequent cross-cultural comparisons shall shed light on delineating the global MSM mixing landscape.
